# Polyhydroxyalkanoate (PHA) Granules Have no Phospholipids

**DOI:** 10.1038/srep26612

**Published:** 2016-05-25

**Authors:** Stephanie Bresan, Anna Sznajder, Waldemar Hauf, Karl Forchhammer, Daniel Pfeiffer, Dieter Jendrossek

**Affiliations:** 1 Institute of Microbiology, University Stuttgart, Germany; 2 Department of Organismic Interactions, Eberhard Karls Universität Tübingen, Germany; 3 Lehrstuhl für Mikrobiologie, Universität Bayreuth, Germany

## Abstract

Polyhydroxybutyrate (PHB) granules, also designated as carbonosomes, are supra-molecular complexes in prokaryotes consisting of a PHB polymer core and a surface layer of structural and functional proteins. The presence of suspected phospholipids in the surface layer is based on *in vitro* data of isolated PHB granules and is often shown in cartoons of the PHB granule structure in reviews on PHB metabolism. However, the *in vivo* presence of a phospholipid layer has never been demonstrated. We addressed this topic by the expression of fusion proteins of DsRed2EC and other fluorescent proteins with the phospholipid-binding domain (LactC2) of lactadherin in three model organisms. The fusion proteins specifically localized at the cell membrane of *Ralstonia eutropha* but did not co-localize with PHB granules. The same result was obtained for *Pseudomonas putida*, a species that accumulates another type of polyhydroxyalkanoate (PHA) granules related to PHB. Notably, DsRed2EC-LactC2 expressed in *Magnetospirillum gryphiswaldense* was detected at the position of membrane-enclosed magnetosome chains and at the cytoplasmic membrane but not at PHB granules. In conclusion, the carbonosomes of representatives of α-*proteobacteria*, β-*proteobacteria* and γ-*proteobacteria* have no phospholipids *in vivo* and we postulate that the PHB/PHA granule surface layers in natural producers generally are free of phospholipids and consist of proteins only.

Polyhydroxybutyrate (PHB) and related polyhydroxyalkanoates (PHAs) are important storage compounds of carbon and energy in *Eubacteria* and *Archaea*. PHB and PHAs are accumulated when cells are living in an environment with a surplus of a suitable carbon source and/or when growth and cell division are impaired due to a limitation by a nutrient other than the carbon source. Research of the last decades in many laboratories has led to a detailed understanding of the biochemical pathways leading to the formation of PHB or PHA granules^1^,^2^,^3^,^4^,^5^,^6^,^7^,^8^. A large number of proteins/genes has been described that are necessary for and are involved in the formation of such storage PHB/PHA inclusion bodies. Detailed descriptions of key enzymes such as PHA synthases, PHA depolymerases and phasins (structural PHA granule associated proteins) are given in numerous publications^9^,^10^,^11^,^12^,^13^,^14^,^15^,^16^,^17^,^18^. Although only three genes are essential to provide the information for the biochemical pathway from the central metabolite acetyl-CoA to the PHB polymer, much more genes with putative functions in PHB metabolism have been identified in *Ralstonia eutropha* H16 (alternative designation *Cupriavidus necator* H16), the model organism of PHB research. The products of these genes comprise the key enzymes of polymerisation (PHB synthases)^19^,^20^,^21^, depolymerisation (PHB depolymerases)^22^,^23^,^24^,^25^,^26^, polymer surface displayed proteins, so-called phasins^27^,^28^,^29^,^30^,^31^,^32^,^33^, and other proteins that are necessary for the regulation and the subcellular localization of the granules^34^,^35^,^36^. Most of these proteins are specifically localized on the surface of PHB granules as was shown by immuno-gold-labelling and transmission electron microscopy^27^,^35^,^37^ or by fluorescence microscopy using fusions of green fluorescent protein variants and the target proteins^26^,^32^,^33^,^38^,^39^,^40^,^41^. The finding of so many proteins on the PHB granule surface with different functions has led to the classification of PHB granules as multifunctional units and the designation as carbonosomes^42^ has been proposed for these organelle-like structures^43^,^44^,^45^. A similar variety of surface-displayed proteins was detected in PHA granules of the model organism of PHA accumulating species, *P. putida*^17^,^46^,^47^,^48^,^49^,^50^ and in other well-studied PHA accumulating species; for a selection see^51^,^52^,^53^,^54^.

However, the exact molecular composition of the PHB/PHA granule surface layer is still not known. While it is generally accepted that several proteins are part of the PHB/PHA granule surface layer *in vivo*, the presence or absence of phospholipids on the PHB/PHA granule surface is controversially discussed^4^,^5^,^8^,^50^,^55^,^56^,^57^. The basis for the assumption that phospholipids are present in the surface layer of PHB granules is the identification of phosphatidic acid and at least one other lipid-like (acetone extractable) compound in purified PHB granules of *Bacillus megaterium* almost 50 years ago^55^ and the detection of phosphatidyl-ethanolamine, phosphatidyl-glycerol, diphosphatidyl-glycerol and a fourth not-identified compound (possibly phosphatidyl-serine) in isolated native PHB granules of *R. eutropha* (at that time designated as *Alcaligenes eutrophus*)^58^. However, it is possible that phospholipids bind artificially to the hydrophobic polymer after disrupture of the cells during the PHB granule isolation process. As a consequence, the identification of phospholipids in isolated PHB granules is not a proof for the *in vivo* presence of phospholipids in the PHB surface layer. Electron microscopy could be a suitable tool to image the surface layer of PHB granules. Indeed, electron micrographs of thin sections of PHB accumulating bacteria were frequently published in the past but the resolution of those images in most cases was of insufficient quality. One example of high technical quality in electron microscopy is the publication of Boatman in 1964 who investigated the inclusions of *Rhodospirillum rubrum* and showed that PHB granules were covered by a surface layer of 4 nm thickness^59^. This value corresponded to approximately half of the size of a cytoplasmic membrane and would be indicative for a (phospholipid) monolayer. If the PHB surface layer was composed of phospholipids, a single unit membrane (half of a double layer) with the polar groups facing to the cytoplasm and the hydrophobic tails pointing to the hydrophobic PHB polymer core would make sense. Similar determinations of the thickness of the PHB granule surface layer were made for *R. eutropha (A. eutrophus)* and other PHA accumulating bacteria by co-workers of Frank Mayer’s lab: they determined a value of about 2.9 to 3.8 nm^60^ and concluded that PHB granules in *R. eutropha* most likely are covered by a monolayer. However, the nature of the monolayer, proteins, phospholipids or a mixture of both, could not be resolved.

Recently, high resolution images of PHB accumulating *R. eutropha* cells were published but the nature of the PHB granule surface layer could not be determined^61^. Evidence for a discontinuous surface layer was provided that would not be in agreement with a continuous phospholipid layer. In another PHB accumulating species, *Caryophanon latum*, a relatively thick PHB granule boundary layer of 14 nm was detected in electron micrographs of thin sections. Globular shaped molecules were present in negatively stained samples of PHB granules liberated from *C. latum* and suggested that the surface layer could consist of proteins only^62^. In summary, none of the above-mentioned contributions was able to prove or disprove the *in vivo* presence of phospholipids in the surface layer of PHB granules.

In this study, we addressed this question by the expression of a set of fusion proteins of fluorescent proteins and the LactC2-domain of lactadherin in *R. eutropha*. Lactadherin is a protein of milk fat that specifically binds to phospholipids *in vivo* via its C2 domain^63^,^64^. Fluorescence microscopical analyses of the cells expressing the fusion proteins revealed that only the cytoplasmic membrane contained phospholipids but not the PHA granules of three model organisms. This finding excluded the presence of phospholipids in PHB/PHA granules *in vivo.*

## Results

### Construction of fluorescent fusion proteins and expression in *E. coli*

**Figure 1:**
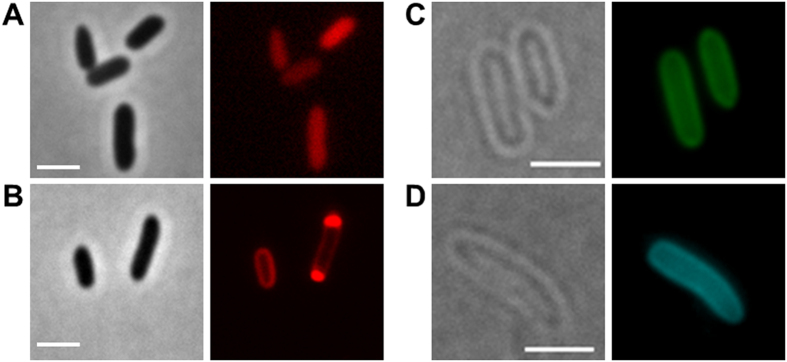
Expression of fluorescent proteins in *E. coli*. (**A**) expression of DsRed2EC alone (phase contrast/red channel), fluorescence visible in the cytoplasm, (**B**) Expression of DsRed2EC-LactC2 fusion (phase contrast/red channel), note, uniform fluorescence of the cell membrane and additional fluorescent foci at the cell poles in some cells, (**C**) Expression of sfGFP-LactC2 fusion (bright field/green channel), (**D**) Expression of mTurquoise2-LactC2 (bright filed/blue channel). Scale bars correspond to 2 μm. Since *E. coli* is not able to synthesize PHB no granules are visible.

Fusions of the fluorescent protein DsRed2EC (or related fluorescent proteins as indicated in Table 1) with the phospholipid-binding domain of bovine lactadherin (LactC2) were constructed and ligated under control of the constitutively expressed promoter of the *R. eutropha phaCAB* operon into the broad host range plasmid pCM62. The expression of fluorescent proteins in *E. coli* (no producer of PHB granules) alone generally resulted in uniform fluorescence and confirmed that the proteins were soluble in the cytoplasm (Fig. 1A). In contrast to this, the cytoplasmic membrane and occasionally the cell periphery near the cell poles were fluorescent when the DsRed2EC-LactC2 fusion was expressed in *E. coli* and only a minor fluorescence signal was detected in the cytoplasm (Fig. 1B). Similar results (localization at the cell membrane) were obtained when fusions of super-folder GFP (sfGFP)^65^ or mTurquoise2^66^ were fused to the LactC2 domain and expressed in *E. coli* (Fig. 1C,D). These results showed that the LactC2 fusion protein was functionally expressed and specifically bound to phospholipid molecules of the cytoplasmic membrane in *E. coli*.

### Expression of DsRed2EC and DsRed2EC-LactC2 in *R. eutropha*

**Figure 2:**
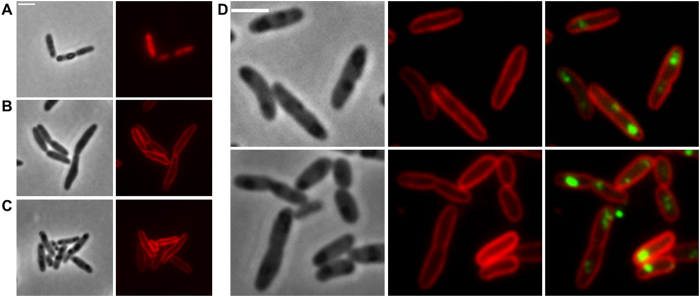
Expression of DsRed2EC and DsRed2EC-LactC2 in *R. eutropha* H16. Expression of DsRed2EC alone in wild type (**A**). Expression of DsRed2EC-LactC2 in ∆*phaC* mutant (**B**) and in wild type (**C**). Phase contrast (left) and red channel (right) in (**A–C**). In (**D**), DsRed2EC-LactC2 was co-expressed with eYFP-PhaC (C319A) in *R. eutropha* wild type (from left to right: phase contrast, red channel, merge of red and green channels). Scale bars correspond to 2 μm.

Next, we transferred the LactC2 fusion constructs to *R. eutropha*. As shown in Fig. 2A, expression of DsRed2EC alone resulted in more or less uniform fluorescence of the cytoplasm and confirmed that DsRed2EC is a soluble protein in *R. eutropha* as in *E. coli*. *R. eutropha* accumulated PHB granules during growth on NB medium. PHB granules can be seen in the phase contrast image of Fig. 2A as dark stained globular structures. Remarkably, no fluorescence was visible in the red channel at the position of the PHB granule. This indicated that DsRed2EC alone is not able to bind to PHB granules.

When we expressed the DsRed2EC-LactC2 fusion first in a ∆*phaC* background (Fig. 2B), in which the cells cannot accumulate PHB granules because of the absence of the key enzyme of PHB biosynthesis (PHB synthase PhaC), a uniform fluorescence of the cytoplasmic membrane was achieved. We conclude that the DsRed2EC-LactC2 fusion specifically binds to the phospholipids of the cell membrane. Next, the DsRed2EC-LactC2 fusion was expressed in *R. eutropha* wild type cells (Fig. 2C). The same uniform fluorescence of the cytoplasmic membrane was observed as found for the ∆*phaC R. eutropha* mutant. However, PHB granules were present and could be detected in the corresponding phase contrast images. The PHB granules appeared as dark regions (in phase contrast) but DsRed2EC-LactC2-specific fluorescence was absent from the suspected positions of PHB granules. As an alternative to identification by phase contrast, PHB granules can be visualized by staining with Nile red but in this case, a differentiation between Nile red and DsRed2EC-LactC2 fluorescence is not possible. To provide a phase contrast-independent proof for the presence of PHB granules in the wild type, PHB granules were labelled by the expression of a fusion of the enhanced yellow fluorescent protein (eYFP) with an inactive PHB synthase (PhaC with C319A mutation) that specifically localizes at the surface of PHB granules. This construct was transferred to *R. eutropha* and expressed together with the DsRed2EC-LactC2 fusion (Fig. 2D). It has been shown previously that the inactive PHB synthase fusion (eYFP-PhaC) was specifically attached to PHB granules in *R. eutropha* without changing the PHB content of *R. eutropha*^33^. It is evident from the overlay images in Fig. 2D that the DsRed2EC-LactC2 fusion did not co-localize with the PhaC-eYFP-labelled PHB granules. The same results were obtained when the mTurquoise2-LactC2 or the sfGFP-LactC2 fusions were expressed in *R. eutropha* wild type in which PHB was stained with the lipophilic dye Nile red (Suppl. Fig. S1). mTurquoise2 and sfGFP have higher fluorescence intensities and shorter folding times, respectively, than DsRed2EC. These results are in line with the presence of phospholipids in the cytoplasmic membrane but contradict the presence of phospholipids on the surface of PHB granules.

### Expression of DsRed2EC-LactC2 in *R. eutropha* phasin mutants

**Figure 3:**
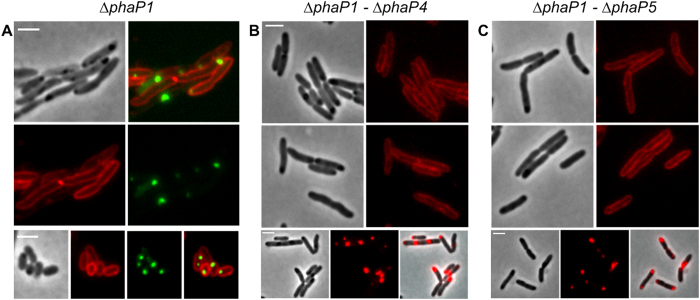
Expression of DsRed2EC-LactC2 in *R. eutropha* phasin mutants. Co-expression of DsRed2EC-LactC2 and eYFP-PhaC in ∆*phaP1* mutant (**A**). Expression of DsRed2EC-LactC2 in ∆*phaP1-*∆*phaP4* mutant (**B**) and in ∆*phaP1-*∆*phaP5* mutant (**C**). Phase contrast and fluorescent images are shown. In the bottom rows of (**B**,**C)** cells were additionally stained with Nile red to indicate the position of PHB granules more clearly than in phase contrast images (phase contrast, red channel, merge). Scale bars correspond to 2 μm.

Phasin proteins, in particular PhaP1, constitute the major fraction of PHB granule associated proteins in *R. eutropha*^26^. To investigate whether the presence of phasin proteins protects the surface layer of PHB granules from interaction with the cytoplasmic membrane and from binding of phospholipids we expressed the DsRed2EC-LactC2 fusion in a ∆*phaP1* background (Fig. 3A), in a ∆*phaP1-*∆*phaP4* background (Fig. 3B) and in a ∆*phaP1-*∆*phaP5* (Fig. 3C) background (in case of the ∆*phaP1* strain with the additional presence of the inactive eYFP-PhaC (C319A) fusion (Fig. 3A)). Essentially, the same results as for the wild type were obtained. A DsRed2EC-LactC2-specific fluorescence could not be detected at the location of the PHB granules in any of the phasin mutant strains; only the cytoplasmic membrane was fluorescent in all cases. These results show that the absence of the major phasin PhaP1 or of any of the phasin proteins PhaP1 to PhaP5 did not enable DsRed2EC-LactC2 to bind to PHB granules. Our data suggest that phospholipids-even in the absence of phasin proteins-apparently do not bind to PHB granules.

### DsRed2EC-LactC2 binds to magnetosome chains but not to PHB granules in *M. gryphiswaldense*

**Figure 4:**
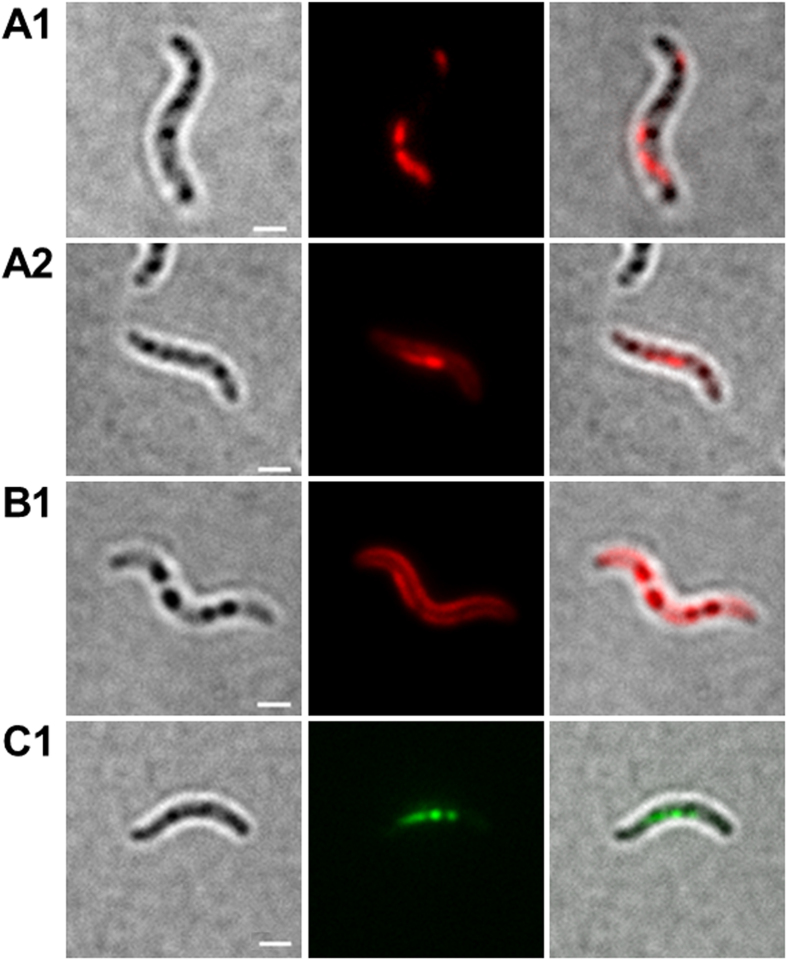
Expression fusion proteins in *M. gryphiswaldense*. Cells expressing DsRed2EC-LactC2 focussed to filament-like fluorescence representing magnetosome-filaments (**A1 and A2)**. Cell expressing DsRed2EC-LactC2 focussed to cell membrane fluorescence (**B1**). Cell expressing MamC-GFP (**C1**). Note, presence of several globular inclusions in all images (PHB granules) that do not co-localize with DsRed2EC-LactC2 or with MamC-eGFP. Individual magnetosomes are too small (≈35 nm) to be visible in bright field. From left to right: bright field, fluorescence channel, merge. Scale bars correspond to 2 μm.

The attachment of phospholipid-binding proteins to membranes could depend on membrane curvature. Since PHB granules are much smaller than whole cells, the curvature of a potential phospholipid membrane of PHB granules would be substantially higher and potentially could prevent binding of LactC2. Moreover, the inner side of the cell membrane has a negative curvature while a potentially existing PHB granule membrane would have a positive curvature. To exclude that binding of the LactC2 domain of lactadherin is specific for a negative membrane curvature we looked for an appropriate positive control of LactC2-binding to membranes with positive curvature. Unfortunately, most inclusions of prokaryotes, as far as known, are not enclosed by phospholipid membranes. Magnetosomes of magnetotactic bacteria, however, are an exception and it is well known that magnetosomes are membrane-surrounded prokaryotic organelles with positive membrane curvature. Notably, magnetosome membranes have a similar composition as the cytoplasmic membrane^43^,^45^. Binding of dsRed-LactC2 to the magnetosome chains of magnetotactic bacteria would be therefore a suited positive control to test the ability of the dsRed-LactC2 fusion to detect membrane-enclosed organelles with a positive membrane curvature. Due to the small size of magnetosomes (≈35 nm in diameter in *M. gryphiswaldense)* in comparison to PHB granules (200–400 nm in diameter) individual magnetosomes cannot be resolved by light microscopy. However, magnetosomes of most magnetotactic bacteria form chains of many magnetosomes aligned on a cytoskeleton-like filament^43^. Therefore, the detection of a filament-like fluorescence signal of DsRed2EC-LactC2 expressed in a magnetotactic species would indicate the binding of the DsRed2EC-LactC2 fusion to the membranes of magnetosomes. We expressed the DsRed2EC-LactC2 fusion in *M. gryphiswaldense* and cultivated the recombinant bacteria under conditions at which they readily form magnetosome chains. The presence of magnetic particles in the recombinant strain was verified by the use of a magnet positioned near the microscope. As shown in Fig. 4A1,A2, chain-like fluorescence signals of recombinant DsRed2EC-LactC2 were visible. These signals resembled images of *M. gryphiswaldense* cells in which the magnetosome-specific MamC protein was fused to eGFP (Fig. 4C1). A non-magnetic *M. gryphiswaldense* mutant strain, in which the 16.4 kbp *mamAB* operon was deleted, did not form any chain-like fluorescent signals when DsRed2EC-LactC2 was expressed (Suppl. Fig. S2). However, DsRed2EC-LactC2 was bound to the cell membrane of *M. gryphiswaldense* cells (for image with focus to cell membrane, see Fig. 4B1). Occasionally, fluorescent foci were detected in the region of the cell membrane or within the cells (Suppl. Fig. S2B1,B2). Since *M. gryphiswaldense* is also able to synthesize PHB and since magnetosomes-containing *M. gryphiswaldense* cells usually harbour one to several PHB granules^67^ it could be that the observed fluorescent foci of DsRed2EC-LactC2 might represent PHB granules to which the fusion protein was attached. However, by comparing the cells shown in Suppl. Fig. S2B1,B2 in bright field and in fluorescence mode it is evident that the dark globular structures (representing PHB granules) did not co-localize with the foci of DsRed2EC-LactC2 that were found in some cells. Examination of other cells with fluorescent DsRed2EC foci never resulted in a co-localization with globular structures visible in bright field. This result corroborated the inability of the DsRed2EC-LactC2 fusion to bind to PHB granules in *M. gryphiswaldense*. Our finding was further verified by the expression of DsRed2EC-LactC2 in a PHB-negative background of *M. gryphiswaldense (*∆*phaCAB)* (Suppl. Fig. S3). Again, chain-like fluorescent signals corresponding to a magnetosome chain were identified in the cells (see Suppl. Fig. S3A1–A4 for cells with focus to chain-like structures). Furthermore, occasionally some cells harboured fluorescent foci (Suppl. Fig. S3B1). Since the ∆*phaCAB* mutant does not produce PHB, the occasionally formed fluorescent foci cannot represent PHB granules. In summary, our data with the *M. gryphiswaldense* strains demonstrate that the DsRed2EC-LactC2 fusion protein was specifically bound to the cell membrane and the membrane-enclosed magnetosome chains but not to the surface layer of PHB granules. These finding are in agreement with the presence of phospholipids in the cell membrane and in the magnetosome membrane but with the absence of such phospholipids in the PHB granule surface layer.

### Expression of LactC2 in PHB accumulating *E. coli*

**Figure 5:**
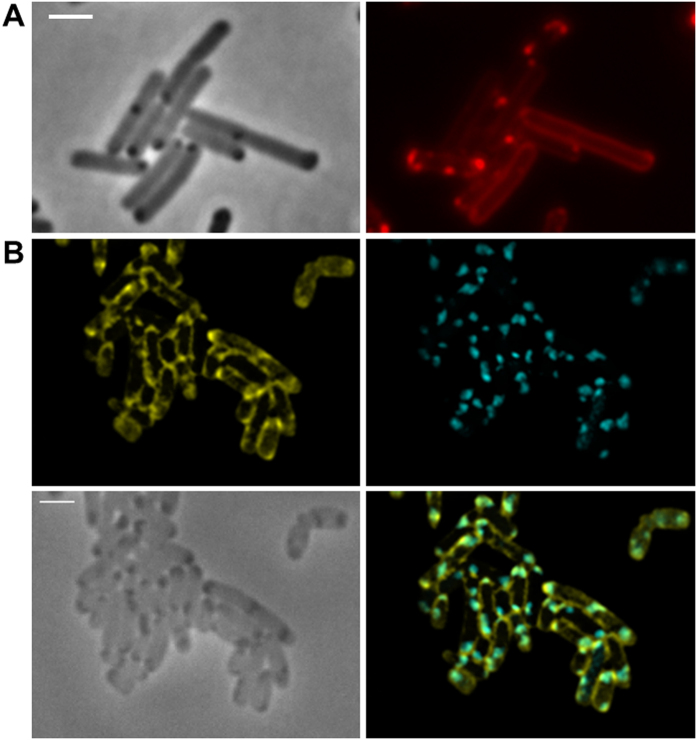
Expression of DsRed2EC-LactC2 and Venus-LactC2 in recombinant PHB accumulating *E. coli*. *E. coli* HMS174 cells co-expressing the *phaCAB* genes of *R. eutropha* (pJM9238) and DsRed2EC-LactC2 are shown in (**A**) phase contrast left, red channel right. Microscopic images of *E. coli* BL21(DE3) co-expressing the *phaC-Cerulean-phaAB* operon and Venus-LactC2 from the pETDuet-vector in (**B**) Venus channel middle left, Cerulean channel middle right, phase contrast bottom left, merge in bottom right. Scale bars correspond to 2 μm.

Previous fluorescence microscopical analysis of recombinant *E. coli* cells that harbour the PHB biosynthetic genes (*phaCAB*) of *R. eutropha* have shown that the first synthesized PHB granules were always located at the cell poles and in the area of the future septum (see Fig. 7 of^68^). Although the resolution of light microscopy did not allow an unambiguous detection it seemed possible that the PHB granules could come into physical contact with the cytoplasmic membrane in a recombinant PHB producing organism. To test if the proximity of the PHB granules to the cytoplasmic membrane in a recombinant *E. coli* strain resulted in the uptake and presence of phospholipids in PHB granules we expressed the DsRed2EC-LactC2 fusion in recombinant PHB accumulating *E. coli* cells. As shown in Fig. 5A, partial co-localization of PHB granules (as localized by phase contrast images) and DsRed2EC-LactC2 fluorescence was indeed observed. However, not all PHB granules revealed a DsRed2EC-LactC2-specific fluorescence. To study the recombinant system in more detail, an *E. coli* strain was constructed in which LactC2 fused to the yellow fluorescent Venus protein and the PHB synthase (PhaC) fused to Cerulean (a brighter CFP variant) were co-expressed from a pETDuet-1 vector. As shown in Fig. 5B, PHB granules-identified by phase contrast and by Cerulean-PHB synthase fluorescence-were indeed preferentially located at the cell poles. Remarkably, we detected an apparent co-localization of some but again not of all PHB granules (PhaC-Cerulean) and Venus-LactC2 fluorescence. These results imply that some recombinant PHB granules in *E. coli* could be covered by lipids. This finding will be discussed below.

### Expression of LactC2-fusions in *P. putida*

**Figure 6:**
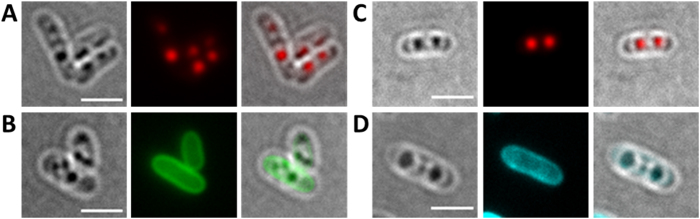
Expression of LactC2 fusion proteins in *P. putida*. Cells of *P. putida* expressing sfGFP-LactC2 (**A**,**B**) or mTurquoise-LactC2 (**C**,**D**) were grown in mineral salts medium with sodium octanoate to promote PHA granule formation. Cells were stained with Nile red in (**A**,**C**) to visualize PHA granules. From left to right: bright field, red (top row) or green/turquoise (bottom row) channel and overlay images. Note, formation of globular inclusions visible in bright field that are stained by Nile-red (PHA granules) but that show no sfGFP-LactC2 or mTurquoise-LactC2 fluorescence. Scale bars correspond to 2 μm.

To determine whether carbonosomes of medium-chain length PHA accumulating bacteria contained phospholipids *in vivo* we transferred the constructs carrying the mTurquoise2- and the sfGFP-LactC2 fusions to *P. putida* and determined the positions of PHA granules and of the fusion proteins (Fig. 6). When the cells were cultivated under PHA permissive conditions (mineral salts medium with sodium octanoate as precursor of PHA building blocks) only the cytoplasmic membrane was fluorescent. PHA granules, that became visible in bright field or after staining with Nile red, showed no LactC2-fusion protein fluorescence. These findings confirmed that medium chain length PHA granules in *P. putida*-similar to short-chain length PHA (PHB) granules in *R. eutropha*-do not contain phospholipids *in vivo*.

### Phosphatidyl-serine decarboxylase localizes at the cytoplasmic membrane and is absent from PHB granules *in vivo*

**Figure 7:**
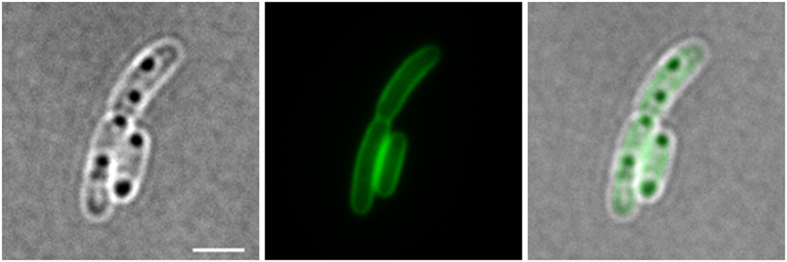
Expression of eYFP-Psd (phosphatidyl-serine decarboxylase) in *R. eutropha* H16. From left to right: bright field, green channel, merge). eYFP-Psd co-localizes with the cell membrane but not with globular structures (PHB granules) that are visible in bright field. Scale bar corresponds to 2 μm.

Phosphatidyl-ethanolamine is the main phospholipid in *R. eutropha* and is synthesized from phosphatidyl-serine by removal of the carboxylic acid group of the serine moiety. This decarboxylase step is catalysed by phosphatidyl-serine decarboxylase that is encoded by the *psd* (A1038) in *R. eutropha*. We constructed an eYFP-Psd fusion and expressed it in *R. eutropha*. Fluorescence microscopical analysis revealed that eYFP-Psd is associated to the cytoplasmic membrane. We assume that the decarboxylation of phosphatidyl-serine to phosphatidyl-ethanolamine is performed shortly before or after the insertion of phospholipids into the membrane. When the strain was cultivated under PHB permissive conditions only a cell membrane-localization of the eYFP-Psd protein was observed and no fluorescence was detected at the position of the PHB granules (Fig. 7). This result is in agreement with the absence of phospholipids in PHB granules.

### Phospholipids bind to PHB granules *in vitro*

As shown above, in PHB accumulating *R. eutropha* cells, fluorescent proteins fused to the phospholipid-binding domain LactC2 of lactadherin were exclusively bound to the cytoplasmic membrane *in vivo.* No evidence for an *in vivo* attachment to the surface layer of PHB granules was obtained. To find an explanation for the *in vitro* detection of phospholipids in isolated PHB granules as described 50 years ago^55^ we purified native PHB granules via two glycerol gradients from *R. eutropha* strains that either expressed DsRed2EC alone or the DsRed2EC-LactC2 fusion and examined the granules by fluorescence microscopy. The PHB granule band of both glycerol density gradients had a white to beige colour and no evidence for the presence of large amounts of DsRed2EC was obtained. When PHB granules isolated from the DsRed2EC expressing strain were examined by fluorescence microscopy, no red-fluorescent granules were detected. This result is consistent with the data of Fig. 1 and confirmed that DsRed2EC has no affinity to PHB granules either *in vivo* or *in vitro*. When PHB granules that had been isolated from the DsRed2EC-LactC2-expressing strain were examined, most PHB granules were also non-fluorescent and showed that the DsRed2EC-LactC2 fusion also has no binding affinity to PHB granules. However, occasionally we detected PHB granules (less than 1% of isolated PHB granules) that showed red fluorescence (Suppl. Fig. S4). This indicated that in some cases the DsRed2EC-LactC2 fusion apparently can bind to PHB granules. We assume that fragments of the cytoplasmic membrane were artificially bound to PHB granules in a minor fraction of PHB granules and that the DsRed2EC-LactC2 fusion was able to bind to these phospholipid-contaminated PHB granules *in vitro*. This finding can explain the detection of trace amounts of phospholipids in isolated native PHB granules half a century ago by Griebel and co-workers^55^.

## Discussion

A variety of phospholipid-binding proteins is known from literature (for overviews see^69^,^70^). Many of them depend on the presence of Ca^2+^ and/or are specific for phospholipids present in eukaryotes such as phosphatidyl-inositols and their mono- or multi-phosphorylated variants^70^,^71^. Blood clotting factor V and factor VIII as well as bovine lactadherin represent two Ca^2+^-independent phospholipid-binding proteins^63^,^72^. These proteins specifically bind to phospholipids via C-terminal located phospholipid-binding domains (C-domains). In lactadherin, two of such binding domains are present (C1 and C2 domain). In particular, the C2-domain (LactC2) is responsible for the high binding affinity of lactadherin to phospholipids^64^. The crystal structure of lactadherin was solved and the membrane binding part has been identified^73^. In an early publication using an *in vitro* binding assay, a low specificity of lactadherin for a variety of phospholipids such as phosphatidyl-serine (PS), phosphatidyl-ethanolamine (PE), phosphatidyl-inositol (PI), phosphatidyl-glycerol (PG), phosphatidic acid and cardiolipine (CL) but not to phosphatidyl-choline (PC) was shown^63^. Later, it was suggested that the LactC2 domain apparently conferred high membrane binding ability in particular to PS-containing membranes *in vivo*^64^,^74^. The extent to which the LactC2 domain binds also to phospholipids other than PS *in vivo* is not exactly known. However, lactadherin binds to a phospholipid mixture with an excess of PE in comparison to the concentration of PS even if the PS content is only 0.03%. Another factor that stimulates binding of lactadherin to PS-containing phospholipids is a high degree of curvature^74^ as it is present near the cell poles of rod-shaped bacteria and in small vesicles and PHB/PHA granules if such granules would contain phospholipids.

PE, PS and CL are the main components in eubacterial membranes. In *R. eutropha*, PG, PE, PS, and CL have been identified as membrane lipids^75^ with PE as the major component. Since lactadherin is able to bind to phospholipids with only trace amounts of PS, the DsRed2EC-LactC2 and related fusion constructs of our study should be suitable tools to detect PS-containing phospholipids *in vivo*. Indeed, a fusion of the LactC2 domain of bovine lactadherin with a green fluorescent protein has been previously used to detect PS-containing membranes in mammalian cells^76^. In this study, the specific fluorescence of the cell membranes of *R. eutropha*, *E. coli*, *P. putida* and of *M. gryphiswaldense* upon expression of the DsRed2EC-LactC2 construct is in full agreement with the presence of phospholipids in cell membranes of these species and confirms the specificity of the fluorescent LactC2 construct for the detection of phospholipids in prokaryotes. The finding that DsRed2EC-LactC2 was able to bind to the magnetosome chains of *M. gryphiswaldense* illustrates that also membranes of prokaryotic organelles with a strong positive curvature can be detected. The nature of the DsRed2EC-LactC2 foci that were observed in a minor fraction of the cell population remains unclear and might indicate the presence of a rare, yet to be identified membrane-embedded structure.

**Figure 8:**
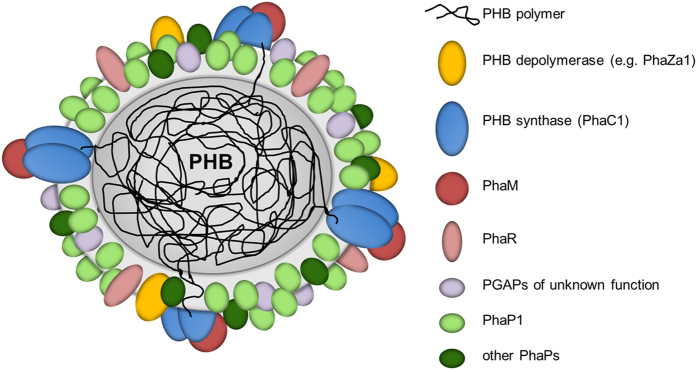
Model of an *in vivo* PHB granule in *R. eutropha* H16. The surface layer is free of phospholipids and consists of proteins only. The presently known PHB granule associated proteins (PGAPs) are symbolised by differentially coloured globules. All proteins in this model had been previously shown to be bound to PHB granules *in vivo* by expression of appropriate fusions with fluorescent proteins. For details and overview see references^8^,^26^. The dimension of the surface layer is enlarged relative to the polymer core for better visibility.

We never observed a co-localization of LactC2 fused to DsRed2EC or of one of the other fluorescent fusion constructs with carbonosomes in *R. eutropha*, *P. putida. or M. gryphiswaldense*. The simultaneous binding of DsRed2EC-LactC2 to the cell membrane and to the magnetosome chain but its absence at PHB granules in *M. gryphiswaldense* strongly indicates that PHB granules apparently do not contain phospholipids in the surface layer *in vivo.* A consequence of our findings is that the surface layer of carbonosomes is a protein layer. A current model of the structure of a PHB granule with all proteins identified *in vivo* for *R. eutropha* is given in Fig. 8.

Only when PHB granules were synthesized in a recombinant *E. coli* background, a potential co-localization of LactC2 was detected for some PHB granules. The artificial *E. coli* system lacks the native surface of carbonosomes as it can be found in *R. eutropha* or *P. putida*^8^,^26^,^49^,^57^. Hence, the hydrophobic surface of the polymer in *E. coli* is at least partially exposed to the cytoplasm where it might come in contact with the cytoplasmic membrane, in particular because PHB granules in *E. coli* tend to localize close to the cell poles. In *R. eutropha* and in *P. putida,* carbonosomes (and possibly also in *M. gryphiswaldense*) are attached to a subcellular scaffold (most likely the bacterial nucleoid) via interaction with PhaM^36^,^77^ and PhaF^50^ and do not localize at the cell poles even in the absence of PhaP1. As a consequence of over-production and close localization to the cell periphery, PHB granules in recombinant *E. coli* might abstract phospholipids to the PHB granule surface. The same artificial binding of phospholipids to the PHB granule surface layer happens *in vitro* during the PHB granule isolation process and was confirmed in this study by the occasional detection of DsRed2EC-LactC2 fluorescence in an isolated PHB granule fraction. This finding well explains the detection of traces of phospholipids in isolated PHB granules by Merrick and co-workers almost 50 years ago^55^. Since no LactC2-conferred fluorescence was detected in PHB or PHA granules in *R. eutropha* (PHB accumulating representative of β*-proteobacteria)*, *P. putida (*PHA accumulating representative of γ*-proteobacteria)*, or *M. gryphiswaldense* (PHB accumulating representative of α*-proteobacteria)*, we conclude that membrane lipids are not present in the surface layer of native PHB/PHA granules *in vivo*. The fact that recombinant PHB granules in a foreign host can be covered by LactC2-reacting material demonstrates that the detection system is fairly sensitive and would detect phospholipids if present. In conclusion, we have ruled out the presence of phospholipids *in vivo* in naturally formed PHB and PHA granules and this is in line with cryo-tomography data^61^. We assume that the finding of phospholipids in isolated PHB granules most likely is an *in vitro* artefact and reflects the potential of carbonosomes to accommodate lipids on its surface under non-physiological conditions. As a further observation of this study, the enrichment of DsRed2EC-LactC2 near the cell poles in some cells might indicate that prokaryotic membranes can have a non-random distribution of phospholipids similar to the presence of so-called lipid rafts.

## Methods

### Bacterial strains, plasmids and culture conditions

Bacterial strains and plasmids used in this study are shown in Table 1. *E. coli* strains were grown in Lysogeny broth (LB) medium supplemented with the appropriate antibiotics at 37 °C. In some cases 0.5% (wt/vol) of glucose was added to promote accumulation of PHB of recombinant *E. coli* strains harbouring the PHB biosynthetic genes. *R. eutropha* H16 strains were grown on nutrient broth (NB, 0.8%, (wt/vol)) with or without addition of 0.2% (wt/vol) of sodium gluconate at 30 °C. *Pseudomonas putida* GPO1 (previously *P. oleovorans*) was grown in mineral salts medium with addition of sodium octanoate (0.3%, wt/vol) at 30 °C. *M. gryphiswaldense* was grown in modified flask standard medium (FSM) at 28 °C in 15 ml polypropylene tubes with sealed screw caps and a culture volume of 10 ml at micro-oxic conditions with moderate shaking (120 rpm)^78^.

### Construction of fluorescent fusion proteins

Constructions of in frame fusion proteins generally were prepared via PCR. The DNA sequences of the used synthetic desoxyribo-oligonucleotides and the amino acid sequences of the resulting fusion proteins are shown in Suppl. Tables S5 and S6. All PCR constructs were ligated into appropriate vectors and transformed to *E. coli*. The DNA sequence of each construct was verified by commercial DNA sequencing of the entire length of the PCR-amplified region and only constructs with correct DNA sequence were used.

The plasmids were transformed to *E. coli* by standard transformation and were subsequently transferred via conjugation from recombinant *E. coli* S17-1 to *R. eutropha* H16. Selection was achieved by plating on mineral salts medium supplemented with 0.2% fructose and 15 μg ml^−1^ tetracycline or 350 μg ml^−1^ kanamycin, respectively. For *P. putida* GPO1, plasmids were transformed using electroporation as described elsewhere^79^. Selection was achieved by plating on NB medium and 15 μg ml^−1^ tetracycline. For *M. gryphiswaldense*, pBAM plasmids were transferred from *E. coli* WM3064 to *M. gryphiswaldense* MSR-1. Selection was achieved by addition of 5 μg/ml kanamycin.

### Isolation of PHB granules

PHB granules were isolated from French press (twice) disrupted cells by two subsequent glycerol gradient centrifugations as described previously^80^.

### Microscopical methods

Formation of PHB granules was followed by fluorescence microscopy using Nile red as dye (1–10 μg/ml DMSO, Nile red solution at 5–40% [vol/vol]). Fluorescence microscopy and detection of fluorescent proteins (eYFP, DsRed2EC, Venus, Cerulean, sfGFP, or mTurquoise2) was performed on a Zeiss Axioplan, Leica DM5500 B microscope or Nikon Ti-E microscope (MEA53100) by using F41-007 Cy3 and F41-54 Cy2 filters for analysis of the PHB granules (Nile red stained) and DsRed2EC and for eYFP analysis, respectively. Venus fluorescence was detected using an excitation filter ET500/20x and an emission filter ET535/30m. Cerulean fluorescence was detected with excitation filter ET436/20x and an emission filter ET480/40m. A specific filter set (excitation, 415/20 nm; emission, 520/60) was used to visualize mTurquoise2 and sfGFP was detected with the aid of a standard filter set (excitation, 500/24 nm; emission, 542/27nm).

Pictures were taken with a digital camera (Hamamatsu Orca Flash 4.0 sCMOS camera and processed with Nikon imaging software. To avoid a potential cross-talk between fluorescence channels images were recorded with and without Nile red. PHB granules could be also visualized by phase contrast or bright field microscopy. To image fluorescent protein fusions 5 μl portions of the sample were immobilized on agarose pads (1% (wt/vol) in phosphate buffered saline) and covered with a coverslip. Images were processed with ImageJ Fiji v1.50c^81^.

## Additional Information

**How to cite this article**: Bresan, S. *et al.* Polyhydroxyalkanoate (PHA) Granules Have no Phospholipids. *Sci. Rep.* **6**, 26612; doi: 10.1038/srep26612 (2016).

## Supplementary Material

Supplementary Information

## Figures and Tables

**Table 1:** Strains, plasmids used in this study.

Strain/plasmid	Relevant characteristic	Source/reference
*Escherichia coli* JM109	cloning strain	
*E. coli* HMS174		82
*E. coli* S17-1	conjugation strain	83
*E. coli* WM3064	conjugation strain	William Metcalf
*E. coli* BL21(DE3)	Heterologous expression of pET Duet vector	84
*Ralstonia eutropha* H16	Wild type	DSMZ 428
*R. eutropha* H16 Δ*phaP1*	Chromosomal deletion of *phaP1*	31
*R. eutropha* H16 Δ(*phaP1-phaP4)*	Chromosomal deletions of *phaP1-phaP4*	31
*R. eutropha* H16 Δ(*phaP1-phaP5)*	Additional deletion of *phaP5* in *ΔphaP1-phaP4* background	32
*Pseudomonas putida* GPO1	*Pseudomonas putida* wild type strain	85
*M. gryphiswaldense* MSR-1 R/S	Wild type	86
*M. gryphiswaldense mamC-egfp*	*mamC-egfp* chromosomal fusion	67
*M. gryphiswaldense* Δ*mamAB*	deletion of *mamAB* operon	87
*M. gryphiswaldense* Δ*phbCAB*	deletion of *phbCAB* operon	67
pJM9238	expression of *phaCAB*	82
pBBR1MCS2	broad host range vector, Km^r^	88
pBBR1MCS2-P_*phaC*_*-eyfp-c1*	universal vector for construction of fusions C-terminal to eYFP under the P_*phaC*_ promoter	32
pBBR1MCS2-P_*phaC*_*-eyfp-*c1-*psd*	N-terminal fusion of Psd of *R. eutropha* to eYFP	this study
pBBR1MCS-2-P_*phaC*_*-DsRed2EC-c1*	universal vector for construction of fusions C-terminal to DsRed2EC under control of the P_*phaC*_ promoter of *R. eutropha*	this study
pCM62	broad host range vector, Tc^r^	89
pCM62-P_*phaC*_-*DsRed2EC*-c1	universal vector for construction of fusions C-terminal to DsRed2EC under control of the P_*phaC*_ promoter of *R. eutropha*	this study
p416	source of LactC2	76
pETDuet-*phaCerulean-phaAB*-*venus-lactC2*	Co-expression of *phaC-*Cerulean-*phaAB* and Venus-LactC2	this study
pCM62-P_*phaC*_-*DsRed2EC*-c1-*lactC2*	plasmid for expression of DsRed2EC-LactC2	this study
pCM62-P_*phaC*_-*mTurquoise2*-c1-*lactC2*	plasmid for expression of *mTurquoise2*-LactC2	this study
pCM62-P_*phaC*_-*sfgfp*-c1-*lactC2*	plasmid for expression of *sfGFP*-LactC2	this study
pBBR1MCS2-P_*phaC*_-*eyfp*-c1-*phaC1*(C319A)	N-terminal fusion of inactive PhaC1(C319A) to eYFP	33
pBAM-P_*mamDC*_-*dsRed2EC*-c1-*lactC2*	plasmid for expression of DsRed2EC-LactC2	this study
pBAM-P_*tet*_-*dsRed2EC*-c1-*lactC2*	plasmid for expression of DsRed2EC-LactC2	this study

Resistance against kanamycin (Km^r^), tetracycline (Tc^r^).
