# Supplementary material for: Polyhydroxyalkanoate (PHA) Granules Have no Phospholipids
Source: Sci Rep. 2016 May 25;6:26612. doi: 10.1038/srep26612 (PMC4879537; doi:10.1038/srep26612)
Supplement: Supplementary Information [file srep26612-s1.pdf]

Suppl. Materials

Polyhydroxyalkanoate (PHA) Granules Have no Phospholipids

Stephanie Bresan[1], Anna Sznajder[1], Waldemar Hauf[2], Karl Forchhammer[2], Daniel Pfeiffer[3] and Dieter Jendrossek[1]

[1]*Institute of Microbiology, University Stuttgart, Germany*

[2]*Department of Organismic Interactions, Eberhard Karls Universität Tübingen, Germany*

[3]*Lehrstuhl für Mikrobiologie, Universität Bayreuth, Germany*

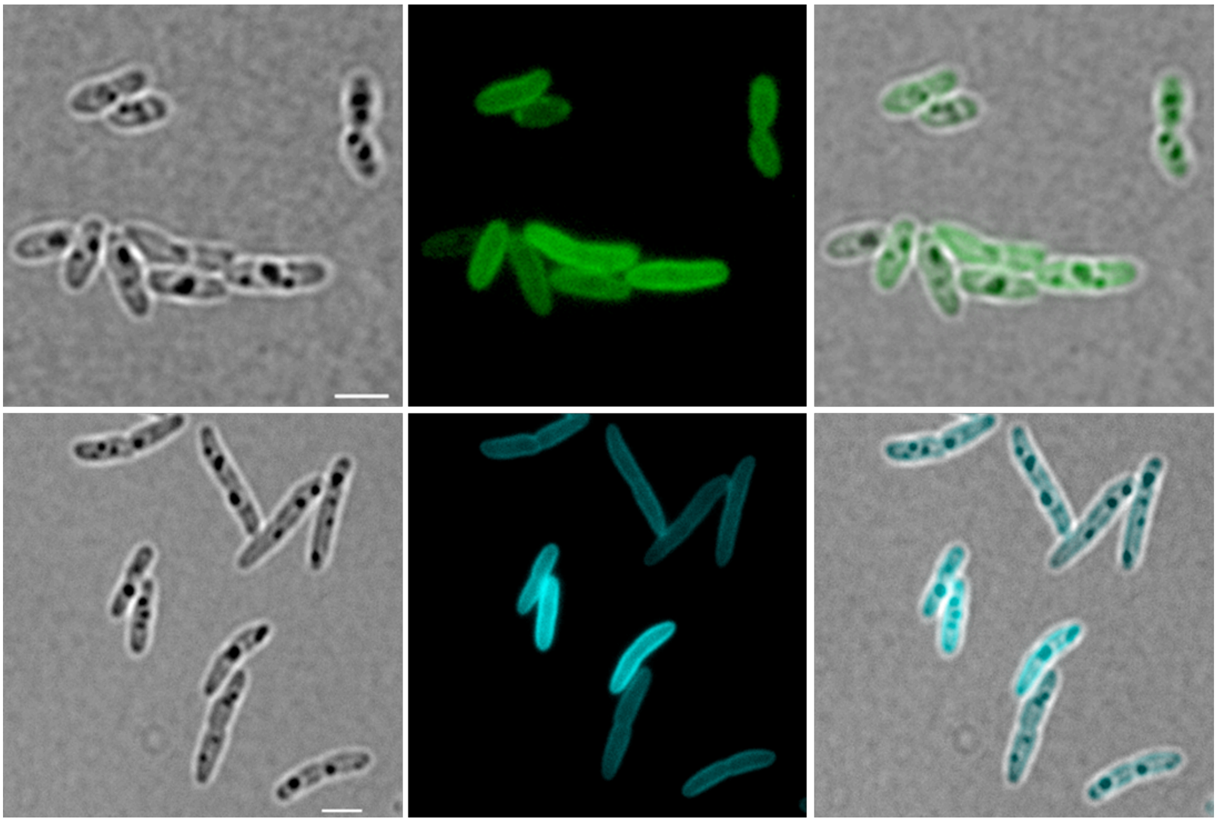

Suppl. Fig. S1: Expression of sfGFP-LactC2 and mTurquoise2-LactC2 in *R. eutropha*.

Cells were grown under PHB permissive conditions. From left to right: bright field, green (top middle) or turquoise (bottom middle) channel, merge. Scale bars correspond to 2 µm. No co-localization of sfGFP-LactC2 or mTurquoise2-LactC2 with PHB granules that are visible in bright field.

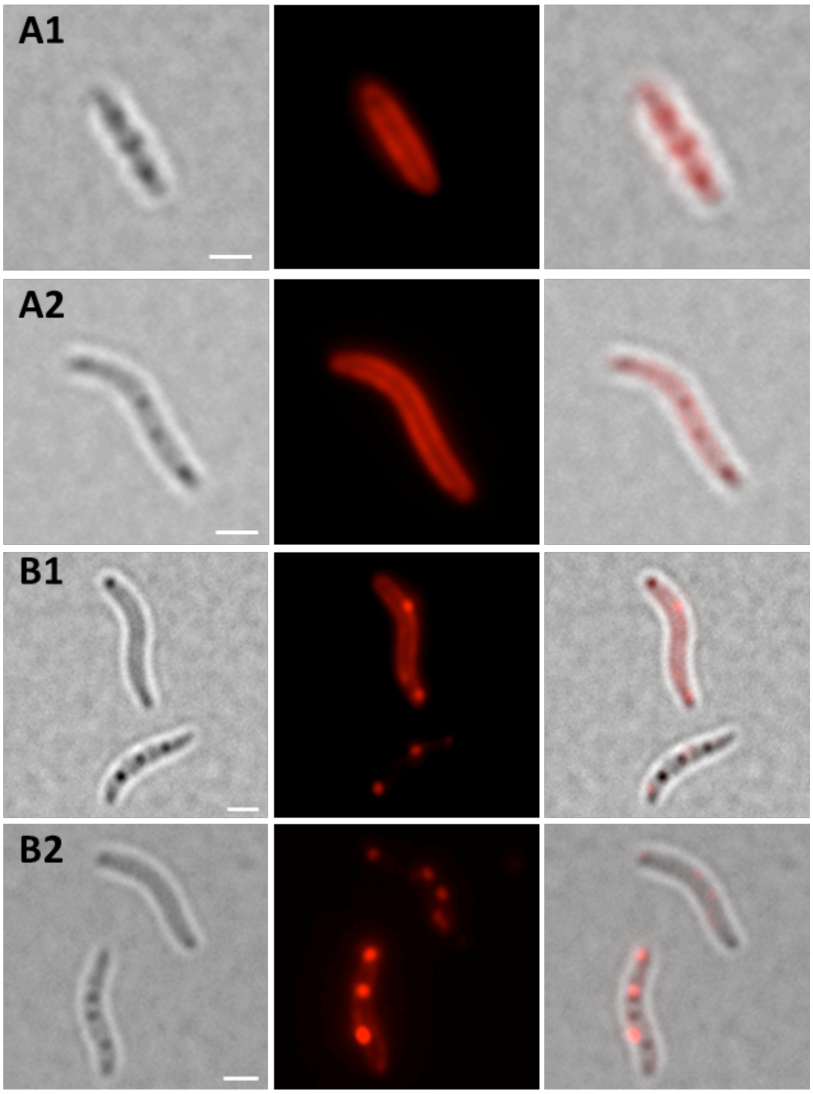

Suppl. Fig. S2: Expression of DsRed2EC-LactC2 in magnetosome-free *M. gryphiswaldense* Δ*mamAB* mutant. Cells showing membrane-bound DsRed2EC-LactC2 fluorescence but no filament-like fluorescence (**A1 and A2**). Cells focussed to foci of DsRed2EC-LactC2 fluorescence (**B1 and B2**). Note, presence of several globular inclusions in bright field (PHB granules) in all images that do not co-localize with DsRed2EC-LactC2 fluorescence. From left to right: bright field, fluorescence channel, merge. Scale bars correspond to 2 µm.

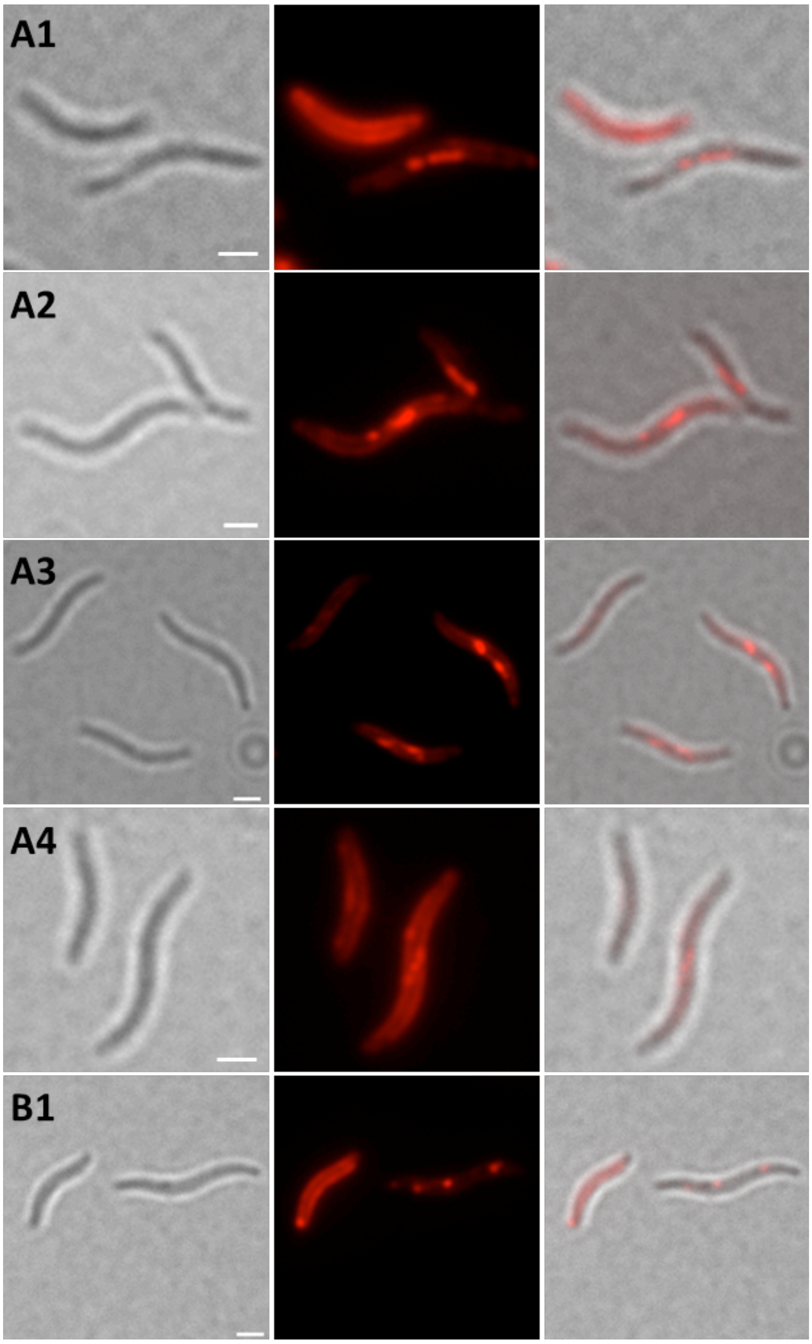

Suppl. Fig. S3: Expression of DsRed2EC-LactC2 in PHB granule-free *M. gryphiswaldense* ∆*phaCAB* mutant. Cells focussed to filament-like dsRed2EC-LactC2 fluorescence resembling magnetosome chains (**A1-A4**). **C**ell focussed to cell membrane-bound DsRed2EC-LactC2 fluorescence and foci of DsRed2EC-LactC2 fluorescence (**B1**). Note, absence of any globular inclusions in bright field (PHB granules). From left to right: bright field, fluorescence channel, merge. Scale bars correspond to 2 µm.

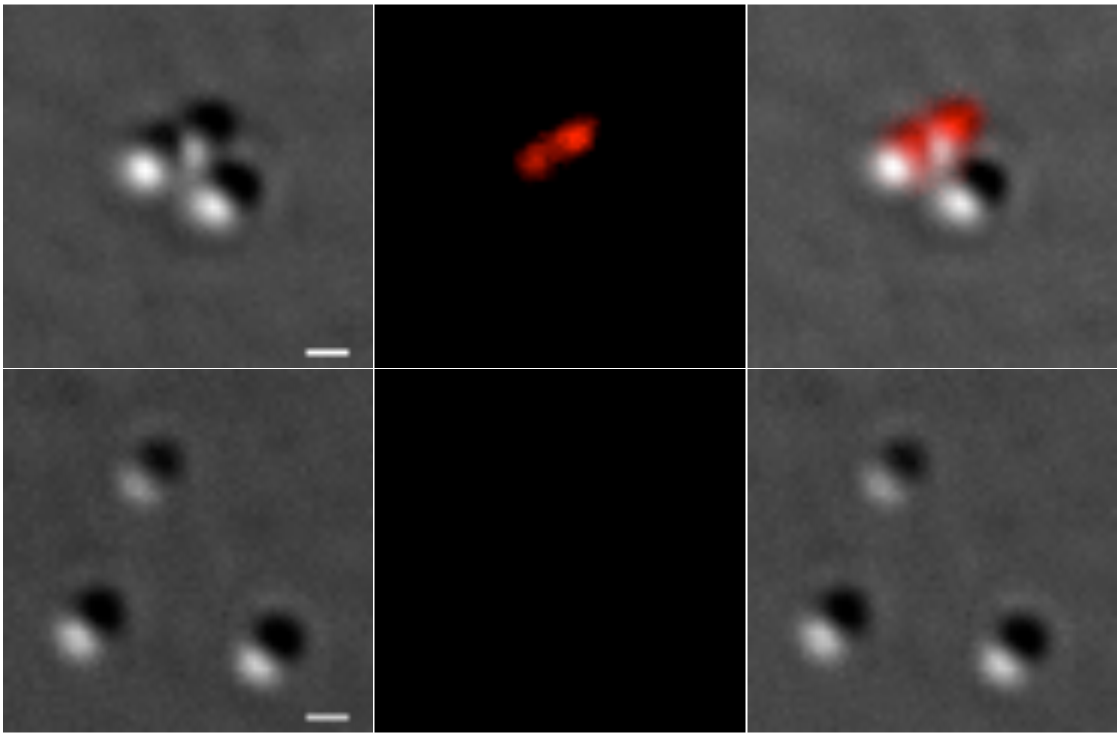

Suppl. Fig. S4: PHB granules isolated from *R. eutropha* H16 cells. PHB granules isolated from *R. eutropha* H16 cells expressing DsRed2EC-LactC2 are shown in the top row. In the bottom row, PHB granules isolated from *R. eutropha* H16 cells expressing DsRed2EC alone are shown. DIC (left), red channel (middle), overlay (right). Scale bars correspond to 0.5 µm.

Suppl. Table S5: Primers used for construction of gene fusions

plasmid	primer for amplification of inserts
pBBR1MCS-2-P_{phaC}-*eyfp*-c1-*psd*	eYFP-Psd-for: ccgctcgaggcatgaactatcctcatccgctgatcgcc<br>eYFP-Psd-rev: cgggatcctcacttcacgtcgagttcggcgaggatg
pCM62-P_{phaC}-*dsRed2*-c1	DsRed2EC-for: gggaattccatatggcgagcagtgagaacatcatcacc<br>DsRed2EC-rev: ggactagttgatcagttatctagatccggtggatcc
pCM62-P_{phaC}-*dsRed2EC*-c1-*lactC2*	LactC2-for: cccaagcttcatgcactgaacccctaggcc<br>LactC2-rev: ggactagtttaacagcccagcagctcc
pETDuet-*phaCcrAB venlactC2*	PhaA-for: gggatcactctcggcatggacgagctgtacaagtgacgcttgcatgagtgccgg<br>PhaB-rev: gaattcggatcctggctgtggtgatgatggtgatggctgctgcctcagcccatgtgcaggccg<br>PhaC- Cerulean-for: cccctctagaaataattttgtttaactttaagaaggagatataccatggcgaccggcaaagg<br>PhaC- Cerulean-rev: gctaccgctgccacttcctgatccgctacctgccttggctttgacgtatcgcc<br>Venus-Lact-for: ccccatcttagtatattagttaagtataagaaggagatatacatatggtgagcaagggcgagg<br>Venus-Lact-rev: cttcaggcctaggggttcagtgcacttgtacagctcgtccatgccgag<br>ReCr-for: gtcaaagccaaggcaggtagcggatcaggaagtggcagcggtagcgtgagcaagggcgaggagctgttc<br>ReCr-rev: ccggcactcatgcaagcgtcacttgtacagctcgtccatg<br>LactC2-for: ctcggcatggacgagctgtacaagtgcactgaacccctaggcctgaag<br>LactC2-rev: gtcagcgatcgcgtggccggccgatatccaattgagatctgcttaacagcccagcagctccac
pCM62-P_{phaC}-*mTurquoise2*-c1-*lactC2*	mmTurq2-LactC2-for: ccatgcaaagtgccggccagggcaatgcccggagccggttcgaatagtgacggcagagagacaa-tcaacatatgagcaagggcgaggagctg<br>mTurq2-LactC2-rev: gggatggtattatccttcaggcctaggggttcagtgcatgaagcttgagctcgagatcttttatatagctcgtccatacccaag
pCM62-P_{phaC}-*sfgfp*-c1-*lactC2*	sfGFP-LactC2-for: ccatgcaaagtgccggccagggcaatgcccggagccggttcgaatagtgacggcagagagacaatcaacatatggcatcaaaaggtgaagaattatttac<br>sfGFP-LactC2-rev: tggtattatccttcaggcctaggggttcagtgcatgaagcttgagctcgagatcttttatataattcatccataccatgtg
pBAM-P_{mamDC}-*dsRed2EC*-c1-*lactC2*	DsRed2EC-LactC2-for: gggaattccatatggcgagcagtgagaacatcatcacc<br>DsRed2EC-LactC2-rev: gggaattcgctagcttaacagcccagcagctccactcg

Suppl. Table S6: Amino acid sequences of constructed fusion proteins

DsRed2EC-LactC2:
MASSENIITEFMRFKVRMEGTVNGHEFEIEGEGEGRPYEGHNTVKLKVTKGGPLPFAWDILSPQFQYGSK
VYVKHPADIPDYKKLSFPEGFKWERVMNFEDGGVATVTQDSSLQDGCFIYKVKFIGVNFPSDGPVMQKK
TMGWEASTERLYPRDGVLKGETHKALKLKDGGHYLVEFKSIYMAKKPVQLPGYYYVDAKLDITSHNEDYT
IVEQYERTEGRHHLFL*RSRAQAS*CTEPLGLKDNTIPNKQITASSYYKTWGLSAFSWFPYYARLDNQGKFNA
WTAQTNSASEWLQIDLGSQKRVTGIITQGARDFGHIQYVAAYRVAYGDDGVTWTEYKDPGASESKIFPG
NMDNNSHKKNIFETPFQARFVRIQPVAWHNRITLRVELLGC

sfGFP-LactC2:
MASKGEELFTGVVPILVELDGDVNGHKFSVRGEGEGDATNGKLTLKFICTTGKLPVPWPTLVTTLTYGVQ
CFSRYPDHMKRHDFFKSAMPEGYVQERTISFKDDGTYKTRAEVKFEGDTLVNRIELKGIDFKEDGNILGHK
LEYNFNSHNVYITADKQKNGIKANFKIRHNVEDGSVQLADHYQQNTPIGDGPVLLPDNHYLSTQSVLSKD
PNEKRDHMVLLEFVTAAGITHGMDELYK*RSRAQAS*CTEPLGLKDNTIPNKQITASSYYKTWGLSAFSWFP
YYARLDNQGKFNAWTAQTNSASEWLQIDLGSQKRVTGIITQGARDFGHIQYVAAYRVAYGDDGVTWTE
YKDPGASESKIFPGNMDNNSHKKNIFETPFQARFVRIQPVAWHNRITLRVELLGC

eYFP-Psd:
MVSKGEELFTGVVPILVELDGDVNGHKFSVSGEGEGDATYGKLTLKFICTTGKLPVPWPTLVTTFGYGLQC
FARYPDHMKQHDFFKSAMPEGYVQERTIFFKDDGNYKTRAEVKFEGDTLVNRIELKGIDFKEDGNILGHK
LEYNYNSHNVYIMADKQKNGIKVNFKIRHNIEDGSVQLADHYQQNTPIGDGPVLLPDNHYLSYQSALSKD
PNEKRDHMVLLEFVTAAGITLGMDELYK*SGLRSRG*MNYPHPLIAREGWPFLAGAFVISLLVHASAGFWW
ALPLWIITVFVLQFFRDPPRPIPSQPNAVLAPADGRIVVVEKTQDPYAGREALKISVFMNVFNVHSNRVSV
DGAVEKVEYFPGKFVNADMDKASVENERNAVLIRRAADGQLVTLVQVAGLVARRILCYTKVGDNLSRGQ
RYGFIRFGSRVDVYLPLDARPRVTIGEKVSASSTILAELDVK

PhaC-Cerulean-PhaAB (sequence of PhaA and PhaB not shown)**:**
MATGKGAAASTQEGKSQPFKVTPGPFDPATWLEWSRQWQGTEGNGHAAASGIPGLDALAGVKIAPA
QLGDIQQRYMKDFSALWQAMAEGKAEATGPLHDRRFAGDAWRTNLPYRFAAAFYLLNARALTELADA
VEADAKTRQRIRFAISQWVDAMSPANFLATNPEAQRLLIESGGESLRAGVRNMMEDLTRGKISQTDESA
FEVGRNVAVTEGAVVFENEYFQLLQYKPLTDKVHARPLLMVPPCINKYYILDLQPESSLVRHVVEQGHTVF
LVSWRNPDASMAGSTWDDYIEHAAIRAIEVARDISGQDKINVLGFCVGGTIVSTALAVLAARGEHPAASV
TLLTTLLDFADTGILDVFVDEGHVQLREATLGGGAGAPCALLRGLELANTFSFLRPNDLVWNYVVDNYLK
GNTPVPFDLLFWNGDATNLPGPWYCWYLRHTYLQNELKVPGKLTVCGVPVDLASIDVPTYIYGSREDHIV
PWTAAYASTALLANKLRFVLGASGHIAGVINPPAKNKRSHWTNDALPESPQQWLAGAIEHHGSWWPD
WTAWLAGQAGAKRAAPANYGNARYRAIEPAPGRYVKAKA*GSGSGSGSGS*VSKGEELFTGVVPILVELDG
DVNGHKFSVSGEGEGDATYGKLTLKFICTTGKLPVPWPTLVTTLTWGVQCFARYPDHMKQHDFFKSAM
PEGYVQERTIFFKDDGNYKTRAEVKFEGDTLVNRIELKGIDFKEDGNILGHKLEYNAISDNVYITADKQKNG
IKANFKIRHNIEDGSVQLADHYQQNTPIGDGPVLLPDNHYLSTQSKLSKDPNEKRDHMVLLEFVTAAGITL
GMDELYK

Venus-LactC2:
MVSKGEELFTGVVPILVELDGDVNGHKFSVSGEGEGDATYGKLTLKLICTTGKLPVPWPTLVTTLGYGLQC
FARYPDHMKQHDFFKSAMPEGYVQERTIFFKDDGNYKTRAEVKFEGDTLVNRIELKGIDFKEDGNILGHK
LEYNYNSHNVYITADKQKNGIKANFKIRHNIEDGGVQLADHYQQNTPIGDGPVLLPDNHYLSYQSKLSKD
PNEKRDHMVLLEFVTAAGITLGMDELYKCTEPLGLKDNTIPNKQITASSYYKTWGLSAFSWFPYYARLDN
QGKFNAWTAQTNSASEWLQIDLGSQKRVTGIITQGARDFGHIQYVAAYRVAYGDDGVTWTEYKDPGA
SESKIFPGNMDNNSHKKNIFETPFQARFVRIQPVAWHNRITLRVELLGC

mTurquoise2-LactC2:

MSKGEELFTGVVPILVELDGDVNGHKFSVSGEGEGDATYGKLTLKFICTTGKLPVPWPTLVTTLSWGVQC
FARYPDHMKQHDFFKSAMPEGYVQERTIFFKDDGNYKTRAEVKFEGDTLVNRIELKGIDFKEDGNILGHK
LEYNYFSDNVYITADKQKNGIKANFKIRHNIEDGGVQLADHYQQNTPIGDGPVLLPDNHYLSTQSKLSKDP
NEKRDHMVLLEFVTAAGITLGMDELYK*RSRAQAS*CTEPLGLKDNTIPNKQITASSYYKTWGLSAFSWFPY
YARLDNQGKFNAWTAQTNSASEWLQIDLGSQKRVTGIITQGARDFGHIQYVAAYRVAYGDDGVTWTEY
KDPGASESKIFPGNMDNNSHKKNIFETPFQARFVRIQPVAWHNRITLRVELLGC

Amino acids coding for the fluorescent protein are indicated by coloured letters, amino acids of the linker region are shown in italic letters and amino acids of the protein of interest are indicated by non-italic black letters
